# Proxy patients’ perceptions of genetic counselor empathy responses

**DOI:** 10.1007/s12687-026-00909-4

**Published:** 2026-06-12

**Authors:** Jennine Bouchard, Ian MacFarlane, Patricia McCarthy Veach, Janice A Baker, Krista Redlinger-Grosse

**Affiliations:** 1 Department of Biochemistry and Medical Genetics, University of Manitoba, Winnipeg, Canada; 2 Department of Genetics, Cell Biology, and Development, Institute of Human Genetics, University of Minnesota, 6-160 Jackson Hall, 321 Church St SE, Minneapolis, MN 55455 USA; 3 University of Minnesota, Minneapolis, USA; 4 Children’s Hospitals and Clinics of Minnesota, Minneapolis, USA

**Keywords:** Genetic counseling, Empathy, Patient perception, Counseling skills

## Abstract

Empathy, the ability to understand and communicate another’s experience to them, positively affects genetic counseling processes and outcomes. Genetic counseling, a specialty healthcare profession, provides education and support for those impacted by genetic disease; thus, empathy is critical. Limited studies have explored patients’ perceptions of genetic counselor (GC) empathy responses. This study investigated the types of GC responses proxy patients perceived as most empathic and why. Female MTurk workers (*n* = 198) completed a survey containing two hypothetical genetic counseling scenarios (Fabry disease; postnatal diagnosis of cleft lip and palate) that ended with a patient statement followed by five different GC responses. Participants were asked to identify the GC response that best conveyed empathy and explain their choice. Every GC response was selected by at least one participant. Inductive content analysis of participants’ rationales yielded six categories: *Understanding*,* Problem Solving*,* Sympathizing*,* Focusing on the Patient*,* Validating*, and *Miscellaneous. Understanding* was the most prevalent rationale for both scenarios (~ 50% each). While GC responses containing the words “I’m sorry…” were chosen most often for both scenarios (~ 45% each), *Sympathizing* was the rationale for only ~ 20% of these GC responses. Logistic regression analyses showed significant predictors varied across scenarios and between rationales, with few significant demographic predictors. Participants differed in preferences for empathy statements and rationales, signaling context and tailoring are important when considering empathy. Patient demographics are not recommended in predicting the type of GC patients may find empathic. Ongoing assessment of patients’ feelings and needs (empathic resonance) will allow genetic counselors to tailor their empathy communication (expressed empathy) accordingly.

## Introduction

Simply defined, empathy is the ability to understand the experience of another person and to communicate that understanding to them (McCarthy Veach et al. 2019). Genetic counseling is a specialized healthcare profession that provides, “Interpretation of family and medical histories to assess the chance of disease occurrence or recurrence; education about inheritance, testing, management, preventions, research and counseling to promote informed choices and adaptation to the risk or condition” (Resta et al. 2006, p. 77). The psychosocial relationship between the counselor and patient within a genetic counseling session is central in the Reciprocal-Engagement Model (REM) of genetic counseling practice, with empathy identified as a critical strategy for building this relationship (McCarthy Veach et al. 2007).

While its use in patient care is well documented and investigated in fields such as psychology and medicine (e.g., Elliott et al. 2018; Jarski et al. 1985; Kim et al. 2004), empirical investigations of empathy in genetic counseling are limited. More research is needed to explore patients’ perceptions of genetic counselor empathy responses. Therefore, the purpose of this study is to examine what types of genetic counselor responses proxy patients perceive as most empathic and why.

### Components of empathy

Empathy can be characterized by a cyclical model based on three components: *empathic resonance*, *expressed empathy*, and *received empathy* (Barrett-Lennard 1981). Empathic resonance involves a person’s ability to sense and understand another individual’s feelings and experience. Expressed empathy refers to the person’s communication of that understanding to the individual. Received empathy concerns the extent to which the recipient of expressed empathy perceives it as communicating understanding. These three components continually repeat during interpersonal interactions. Empathic resonance and expressed empathy vary on a continuum from primary empathy to advanced empathy (McCarthy Veach et al. 2019). Whereas primary empathy focuses on initial understanding and communication of another individual’s surface feelings and experience, advanced empathy is more interpretive, indicating understanding and communication of deeper, less obvious feelings and experience (McCarthy Veach et al. 2019).

### Select research on empathy in health care fields

Research in various healthcare professions suggests empathy is a key component of the patient-provider relationship, though ways of classifying components of empathy differ somewhat across fields. As empathy is multifaceted, there are a variety of ways to define and measure empathy, some of which are summarized below (see Batson 2009 and Gladstein 1987 for a more elaborate discussion). Bachelor and Freedheim (1988) determined four styles of psychotherapy client-perceived empathy. *Cognitive empathy* refers to client perceptions of the accuracy of the therapist’s empathy. *Affective empathy* involves a perception that the therapist is participating in the client’s ongoing emotional state. *Sharing empathy* refers to client perceptions that the therapist discloses personal experiences or opinions, and *nurturant empathy* involves perceptions that the therapist provides a “supportive, security providing, or totally attentive, presence” (p. 232). Using concept analysis, Kunyk and Olson (2001) discerned five components of empathy in nursing literature: empathy as a human trait; empathy as a professional state; empathy as a communication process; empathy as caring; and empathy as a special relationship. Bylund and Makoul (2005) focused on empathic opportunities (“a patient’s explicit statement of emotion,” p. 127), finding such opportunities occurred in every patient-physician interaction and responding to these opportunities elicited further expression of emotion by the patients.

The patient’s perception of empathy is a strong predictor of outcomes, as seen in studies of psychotherapists (e.g., Elliott et al. 2018) and physicians (Jarski et al., 1985; Kim et al. 2004; Wu et al. 2022). For example, in the physician studies, correlations were stronger when physicians used affective empathy statements compared to Bachelor and Freedheim’s (1988) other styles. In their meta-analysis of 82 psychotherapy studies examining therapist empathy and patient outcomes, Elliott et al. (2018) found received empathy (i.e., patient’s perceptions) were the best predictors of therapy outcomes.

### Empathy in genetic counseling

Genetic counseling research on empathy focuses on counselor expressed empathy and its effects on genetic counseling processes and outcomes. Duric et al. (2003) investigated the effects of expressed empathy (defined as a response to content or feeling cues) in 111 hereditary breast cancer genetic counseling sessions. When genetic counselors responded empathically to emotional distress cues, they elicited more emotional distress responses from the patients. They further found patients who received a higher number of counselor empathy responses had greater improvements in post-genetic counseling depression scores than those who received few or no empathic responses. Ellington et al. (2011) analyzed 177 simulated prenatal and cancer genetic counseling sessions and found close to 26% of the genetic counselors’ verbalizations were devoted to client emotional processing. They defined counselor verbalizations relating to client emotional processing as “psychosocial questions, provid[ing] psychosocial information, reassurance, partnering, self-disclosure, concern, [and] empathy” (p. 670).

Nonverbal expressions of empathy have also been examined in genetic counseling and have suggested that more empathically perceived nonverbals correlate with patient outcomes that include satisfaction (Roter et al. 2006). Examples of nonverbal behaviors include (but were not limited to) head nods, eye contact, body lean or positioning, pauses and silence. More recently, Meredith et al., (2025) examined how verbal and nonverbal expression of empathy influence proxy patients’ perception of empathy and effectiveness in genetic counseling. Proxy patients rated empathic nonverbal cues as most effective overall, but in affect reflection statements, neutral nonverbals were preferred; suggesting the effectiveness of nonverbal cues may depend on verbal empathy statements.

Limited studies have looked at patients’ perceptions of empathy in genetic counseling. Tomozawa, Keneko, Sasaki and Miyake (2023) conducted 10 simulated genetic counseling sessions with subsequent interviews with genetic counselor and clients using interpersonal process recall (IPR) to examine their experience of empathy in the simulated sessions in Japan. Clients experiences of perceived empathy were found to increase clients’ self-awareness and provide reassurance and reduction of nervousness. The patient experiences of genetic counseling patients from Japan were further examined in a retrospective interview study that found empathy was perceived as understanding and commitment to the patient (Tomozawa et al. 2025).

These studies suggest empathy is a prevalent and important part of genetic counseling sessions. More research is needed, however, to determine its effects on genetic counseling processes and outcomes, especially patients’ perceptions of different types of counselor responses intended to express empathy.

### Purpose of the study

To date, limited studies have investigated patients’ perceptions of genetic counselor responses specifically intended to express empathy (hereafter referred to as *counselor-defined empathy*). To begin to address this gap, the present study investigated what types of genetic counselor responses proxy patients perceived as most empathic and why. The major research questions were: (1) What types of genetic counselor-defined empathy responses do proxy patients perceive as most empathic? (2) What are proxy patients’ rationales for these choices? and (3) Are there differential effects in rationales based on proxy patient demographics (e.g., age)?

## Method

The University of Minnesota Institutional Review Board (IRB) determined this study met the criteria for exemption of IRB review (STUDY0007520).

## Participants and procedures

The population of interest was genetic counseling patients. As access to actual genetic counseling patients can be challenging, proxy patients were recruited through Amazon’s Mechanical Turk (MTurk), a marketplace website where individuals elect to complete tasks including surveys in exchange for payment. MTurk workers are more diverse than traditional subject pools such as undergraduate students (Goodman et al. 2013; Goodman and Paolacci 2017) and repeatedly produce high quality, reliable data (Goodman et al. 2013; Paolacci and Chandler 2014).

Inclusion criteria for this study were individuals residing in the United States who self-identified as female and ≥ 18 years of age. Only individuals who met the inclusion criteria were able to view the study invitation. The invitation described the research as an investigation of genetic counselors’ empathy responses and contained a link to an anonymous Qualtrics survey. Upon completion of the survey, each participant received a validation code and was compensated $0.40. An a priori power analysis determined a sample size of 215 would be required to detect a moderate effect size (*OR* = 0.65; Cohen 1988) with 0.80 power, but budgetary restraints limited recruitment to 200 (0.80 power detectable *OR* = 0.64 per sensitivity analysis). The survey met the sample quota on a single day in October, 2019. An MTurk technological error resulted in the loss of two participants, leaving 198 who completed the survey.

### Instrumentation

The survey was comprised of two major sections: hypothetical genetic counseling scenarios and demographic questions. Section  1 consisted of two hypothetical genetic counseling scenarios randomly counterbalanced to mitigate possible order effects. Each scenario was preceded by a definition of genetic counseling adapted from the NSGC scope of practice (National Society of Genetic Counselors, n.d.): “Genetic counseling is the process of providing information and support to families who may be at risk for a variety of genetic or inherited conditions” and a brief description of why the patient was seeing a genetic counselor. A definition of empathy was also provided: *Empathy is fully experiencing another person’s feelings and situation and being able to communicate one’s understanding of another’s feelings and experience.*

The patient scenarios and genetic counselor empathy responses are from Kao (2010) who investigated genetic counselors’ written empathic responses to patient statements for five hypothetical genetic counseling scenarios. Kao’s scenarios involved slightly modified patient statements taken from qualitative studies published in the *Journal of Genetic Counseling* between 2007 and 2008. Two of the five scenarios were used in the present study – Fabry disease (Gibas et al. 2008) and Cleft Lip and Palate (Nusbaum et al. 2008). These scenarios were selected because they are consistent in length and both involve female patients. Arguably, the present proxy patient participants would be better able to identify with female patients.

The Fabry disease scenario begins with a brief context and patient statement from Kao (2010, p.160), as follows:

In the scenario you are about to read, Sarah, an adult female, is seeing a genetic counselor and describes her experience living with an inherited condition called Fabry disease. Fabry disease is a genetic condition that causes episodes of extreme pain in the hands and feet and also causes kidney, heart, skin, and vision problems. She is coming in to discuss the chance that her family members may also have this condition. Sarah describes living with the pain associated with Fabry disease and says to the genetic counselor: *“The episodes of pain in my hands and feet are impossible to describe because the pain is so personal and out of control when it comes… The severe abdominal cramping is a sudden onset pain that is scary*,* and something you‘re not sure you can survive. It actually feels like it might kill you. Many times I have passed out – I just hope and pray if I pass out the pain will be over when I come back… if I come back.”*

The Cleft Lip and Palate scenario (herein referred to as the Cleft scenario) begins with the following brief context and patient statements from Kao (2010, p.159), as follows:

Cleft lip and palate are birth defects that occur when a baby’s lip and mouth do not come together properly during pregnancy, but can be successfully repaired with surgery. In the scenario you are about to read, Olivia is seeing a genetic counselor because she recently had a baby who was born with a cleft lip and palate. She is coming in to discuss a possible genetic or inherited cause for her baby’s cleft lip and palate. Olivia describes finding out at birth that her baby was diagnosed with a Cleft lip and palate. She says to the genetic counselor: “*The first words I heard from the doctor were*,* ‘Oh*,* my’… They said it was something that was fixable and not something to be majorly worried about. I didn‘t quite like it at all. I heard the ‘Oh*,* my*,*’ my husband fainted*,* and I thought something was majorly wrong. I actually thought maybe the baby was dead or something.”*

After each scenario, participants were provided with five statements made by different genetic counselor participants in Kao’s (2010) study. Kao asked her participants to write an empathy response to the patient in each scenario. Informed by the Helping Skills Verbal Response System (HSVRS) which classifies types of helper basic skills (Danish, D‘Augelli & Brock, 1976), she coded participant responses into domains reflecting discrete basic skill types.

The verbatim GC responses chosen for each scenario in the present study contain the basic skills with the highest domain prevalence for each scenario in Kao’s investigation. These GC responses are listed below. Of note, the GC responses sometimes included more than one type of basic skill. For reader clarity, the portion of a GC response illustrating the most prevalent domain is italicized. Italics were not included in the actual survey; thus, participants saw the full statements described below.

Fabry Disease scenario.

- Affect response: “*It sounds like your pain makes you feel out of control and helpless*.”
- Content response: “*It sounds like the pain associated with this condition has had a severe impact on your life and it is something that you think about very often….”*
- Influencing response: “*Pain management can be quite difficult*,* especially when you don’t know when the pain will start*,* or where it will be.* It must make your life very difficult and unpredictable.”
- Question response: “That type of pain must be very difficult to live with and to never know when it may come, that must very unsettling. *Are there times you just wish you’d pass out?”*
- Self-involving response: “*I’m very sorry to hear that you are in such pain. It’s hard for me to imagine these episodes that you describe.* I would like to try to help you find a physician/pain management group to help you.”

Cleft scenario.

- Affect response: *“You were really scared to find out what the doctor was talking about.”*
- Content response: “*When you heard that initial reaction from the doctor*,* it made you feel something was very wrong.* Hearing the news that way was upsetting.”
- Influencing response: *“Sometimes doctors and those present in the room react to what they are seeing without thinking about how their reactions will affect you.”*
- Question response: “That must have been difficult, *tell me what happened next.*”
- Self-involving response: “*I’m so sorry that you had that experience with the birth of your child.* Hearing those words from the mouth of the doctor must have been awful.”

The order of the statements was randomly counterbalanced across participants in the present study. Participants were asked “Which of the following responses BEST conveys the genetic counselor’s understanding. Next, they were asked in an open text box, “Why do you think the response you chose communicates the best understanding of the patient?” Participants repeated this process for both scenarios.

The second section of the survey contained demographic questions (see Table 1). Four additional questions asked whether participants had heard of the term genetic counseling prior to this survey; if yes, how familiar were they with genetic counseling (1 = *Little/No Familiarity*, 4 = *Very Familiar*); and whether they had a personal or family history of Fabry disease or Cleft lip and palate.

**Table 1:** Respondent demographics (*N* = 198)

Variable	M		Mdn	SD	Range	
Age	39.4		36	13.1	20–79	
# of People Living in Household	3.0		3.0	1.3	1–9	
**Variable**	***n***	**%**	**Variable**		***n***	**%**
Racial Identity			Heard the Term GC			
White	139	70.0	Yes		131	66.2
Asian	24	24.5	No		51	25.8
Black/African American	14	7.1	Not sure		16	8.1
Hispanic/Chicano/Lationa(o)	12	6.1	How Heard of GC			
Bi-racial	6	3.0	Media		44	22.2
American Indian or Alaskan Native	2	1.0	Participation in GC^a^		25	12.6
Other	1	0.5	Family/Friend		14	7.1
Highest Leve of Education			Class/Academic Setting		13	6.6
Some high school	3	1.5	Other^b^		7	3.5
High school graduate/GED	9	4.5	Familiarity with GC			
Some college	47	23.7	Little/No Familiarity		85	42.9
Associate’s degree	29	14.6	Somewhat Familiar		83	41.2
Bachelor’s degree	77	38.9	Familiar		19	9.5
Master’s degree	29	14.6	Very Familiar		11	5.6
Professional school degree	3	1.5	Offered Genetic Testing			
Doctorate	1	0.5	Yes		67	33.8
Relationship Status			No		124	62.6
Legally Married	94	47.4	Not sure		7	7.1
Single	45	22.7	Who Offered Genetic Testing			
Long-term relationship/Partnered	25	17.7	Doctor		50	74.6
Divorced	22	11.1	Self		9	13.4
Widowed	1	0.5	Genetic Counselor		5	7.5
Have Children			Other^c^		3	4.5
Yes	117	59.0	Family History^d^: Fabry disease			
No	78	39.3	Yes		11	5.6
Annual Household Income			No		182	91.9
< $10, 000	12	0.6	Not Sure		5	2.5
$10,000-$14,999	7	0.4	Family History^d^: Cleft Lip/Palate			
$15,000-$24,999	16	8.1	Yes		16	8.1
$35,000-$49,999	20	10.1	No		179	90.4
$50,000-$74,999	32	16.2	Not sure		3	1.5
$75,999-$99,999	46	23.3				
$100,000-$149,999	32	16.2				
$150,000-$199,999	22	11.1				
≥ $200,000	1	0.5				
Prefer not to answer	5	2.5				

Note. GC= genetic counseling; ^a^Either self or knowing someone who participated; ^b^Includes work, doctor, military to identify remains, articles; ^c^directly from a company, website; ^d^ Includes personal and/or family history

The survey was piloted independently with a genetic counselor and two individuals who had no genetic counseling background, resulting in minor revisions to clarify a few items.

### Data analysis

#### **Qualitative data**

Open-ended responses were analyzed using inductive content analysis (ICA; Vears and Gillam 2022) to identify common categories in participants’ reasons for their preferred genetic counselor statements. ICA is an inductive approach for describing conceptually similar content in responses. JB, a genetic counseling graduate student at the time of the study, initially categorized responses into categories and assigned each category a label reflecting the concept contained therein. Responses were coded in entirety (i.e., not segmented), although if responses fit multiple categories, they were coded as such. JB and KRG, a licensed psychologist and certified genetic counselor with research expertise in qualitative analysis, discussed shared understanding of the coding process and final classifications. Any disagreements were discussed until consensus was determined. Coding was done iteratively as categories were refined and until no additional response categories (i.e., rationales) emerged.

#### **Quantitative data**

All analyses were conducted using R version 3.6.2 (R Core Team, 2019). Descriptive statistics were calculated for survey responses. Consistency of participant response rationales were assessed using McNemar’s test, which accounts for the repeated-measures nature of the data. Logistic regression analyses were conducted to predict the rationales provided by participants for the GC responses they selected. As some participant responses were multifaceted and thus were classified within multiple categories, a separate regression analysis was conducted to predict each rationale. We used a backward stepwise approach to determining the optimal model to interpret, using the Akaike Information Criterion (AIC) as the stopping criterion (Kunter et al., 2005). There are numerous potential criteria to use to determine the final model (e.g., predictor *p*-values, partial correlations, *R*^2^). We chose AIC as it prioritizes parsimonious models (Kunter et al., 2005), which can be particularly useful in early exploratory studies. The numerical value of a model’s AIC is not directly interpretable, but smaller numbers represent better model fit. The stepwise method we followed identified the model with the smallest possible AIC using our predictors. The initial model contained demographic and GC response variables and subsequent models removed the predictor which would provide the largest reduction in AIC. The process continued until no variables would produce a reduction in AIC. We did not require a statistically significant ΔAIC, as we sought the most parsimonious model. *Problem solving* was not included for the Cleft scenario as only a single participant provided that rationale which is not sufficient to run the model. We also did not analyze *Miscellaneous*, as the responses were not a unified rationale.

## Results

### Sample demographics

Table 1 contains a summary of respondents’ demographics. The sample was 70% white, with a mean age of 39.4 (*SD* = 13.1; Range: 20–72). A majority (79.2%) had at least some post-secondary education and had heard the term *genetic counseling* (62.0%). Overall familiarity with genetic counseling was low, however, with 42.9% reporting little to no familiarity, and 41.2% reporting being somewhat familiar. Few participants indicated a personal or family history of Fabry disease (5.6%) or Cleft Lip/Palate (8.1%).

### Preferences for GC empathy responses for each scenario

The genetic counselor empathy statements and the rates at which they were selected are shown in Table 2. Every GC response was selected as the best by at least one participant. The most commonly selected GC response (*n* = 86) for the Fabry disease scenario was self-involving: “I’m very sorry to hear that you are in such pain. It’s hard for me to imagine these episodes that you describe. I would like to try to help you find a physician/pain management group to help you.” Next more prevalent was influencing (*n* = 44): “Pain management can be quite difficult, especially when you don’t know when the pain will start, or where it will be. It must make your life very difficult and unpredictable.” Self-involving (*n* = 87) was the most commonly selected GC response for the Cleft scenario: “I’m so sorry that you had that experience with the birth of your child. Hearing those words from the mouth of the doctor must have been awful.” Next most prevalent (*n* = 51) was content: “When you heard that initial reaction from the doctor, it made you feel something was very wrong. Hearing the news that way was upsetting.”

**Table 2:** Frequency of GC responses selected by proxy patients and rationale categories for fabry disease and cleft lip and palate scenarios

Response	*n*	Frequency of Response Rationale Categories^a^					
		Understanding	Sympathy	Problem Solving	Focusing on Pt.	Validating	Misc.
**Fabry disease** *I’m very sorry to hear that you are in such pain. It’s hard for me to imagine these episodes that you describe. I would like to try to help you find a physician/pain management group to help you.* (Self-Involving)	86	34	15	57	1	17	11
*Pain management can be quite difficult*,* especially when you don’t know when the pain will start*,* or where it will be. It must make your life very difficult and unpredictable.* (Influencing)	44	30	0	5	3	8	6
*It sounds like the pain associated with this condition has had a severe impact on your life and it is something that you think about very often…* (Content)	39	21	2	1	5	3	10
*That type of pain must be very difficult to live with and to never know when it may come*,* that must very unsettling. Are there times you just wish you’d pass out?* (Question)	16	10	1	0	2	1	4
*It sounds like your pain makes you feel out of control and helpless.* (Affect)	14	5	0	3	1	1	5
**Cleft Lip and Palate** *I’m so sorry that you had that experience with the birth of your child. Hearing those words from the mouth of the doctor must have been awful.* (Self-Involving)	87	54	19	0	6	24	9
*When you heard that initial reaction from the doctor*,* it made you feel something was very wrong. Hearing the news that way was upsetting.* (Content)	51	33	3	0	0	11	11
*That must have been difficult*,* tell me what happened next*. (Question)	29	7	2	0	14	5	7
*Sometimes doctors and those present in the room react to what they are seeing without thinking about how their reactions will affect you.* (Influencing)	21	3	0	1	0	3	15
*You were really scared to find out what the doctor was talking about*. (Affect)	10	1	0	0	0	1	8

Note. GC = Genetic Counselor. ^a^Some participants provided rationales for a GC response that were multifaceted and therefore were coded as more than one theme, thus numbers may not sum across rows

### Respondents’ rationales for their preferred GC responses

Inductive content analysis of participants’ rationales for why they selected a specific GC response for each scenario yielded six categories: *Understanding*,* Problem Solving*,* Sympathizing*,* Focusing on the Patient*,* Validating*,* and Miscellaneous*. Some participants provided rationales that were multifaceted, which were coded to more than one empathy response theme (see Table 2 for frequency of rationale codes and Table 3 for illustrative examples). These categories were identified in both scenarios. *Understanding* includes participant statements that the genetic counselor conveyed an overall recognition of the patient’s experience and/or feelings. *Problem Solving* refers to statements that the counselor conveyed a willingness to help, fix, or find a solution for the patient. *Sympathizing* refers to statements that the counselor expressed compassion for what the patient had experienced. *Focusing on the Patient* pertains to statements that the genetic counselor directed attention in the conversation to the patient. *Validating* refers to statements that the counselor provided support for the patient’s feelings. *Miscellaneous* consists of response which could not otherwise be classified within the other categories.

**Table 3:** Frequency of categories for participants’ rationales for the GC response option they chose for each scenario and illustrative quotes

Theme	Fabry Scenario	*n*	Cleft Scenario	*n*
Understanding	*It acknowledges the patient’s complaints and it also shows an understanding of seeing things from the patient’s perspective.* *Makes it sound like counselor understands both the level of pain and the impact on the life situation.*	100	*It shows the person can understand how it would feel to have that experience.* *It shows the genetic counselor was paying attention to what I said and understands my pain.*	98
Problem Solving	*Because the counselor cannot relate to the pain herself*,* explains this*,* and offers Sarah assistance in finding someone who might be able to help her manage that pain during episodes.* *It conveys that the counselor has actually listened*,* is concerned*,* and wants to help.*	66	*It gives the patient a plausible answer to fall back on.*	1
Sympathizing	*It’s the most sympathetic. A little sympathy and understanding can really help people that are suffering.* *B* *ecause they are being sympathetic to the patients’ symptoms.*	18	*It’s nice to apologize for the experience that she had since she got very scared accidentally by the doctor.* *It’s very apologetic and the most sympathetic response towards the bad experience the couple had with the doctor.*	24
Focusing on the Patient	*This invites the patient to talk more about their pain.* *It is an open-ended question and invites the patient to continue speaking about her feelings.*	12	*They acknowledge the difficulty and encourage the patient to continue.* *She is open to hearing more about the mother’s feelings and fears.*	20
Validating	*The genetic counselor is affirming what the patient said and acknowledging their pain.* *It acknowledges how the patient must be feeling. It validates her suffering.*	30	*She described the initial response from the Dr. & the counselor acknowledges this and that the news of cleft palate was upsetting because it was not the best way to deliver it to the parents.* *Validation without trivializing her experience or justifying the other doctor’s response.*	44

Note. GC = Genetic Counselor. *n*’s refer to the number of statements. Some participants provided rationales that were multifaceted and therefore they were coded as more than one theme

For both the Fabry and Cleft scenarios, *Understanding* was the most prevalent category (*n =* 100 and 98 responses, respectively). *Problem Solving* was a predominant category for the Fabry scenario (*n =* 66 responses), but it was coded only one time for the Cleft scenario. McNemar’s test to assess consistency of rationale provided across scenarios found no significant differences in the use of *Understanding*,* Sympathizing*, and *Focusing on the Patient* by scenario, indicating participants who used these rationales consistently applied them across scenarios. *Problem Solving* was reported at a higher frequency in the Fabry scenario (*p*<.001), while *Validating* (*p*=.05) and *Miscellaneous* (*p*=.04) were reported more frequently in the Cleft scenario, indicating participants did not use these rationales consistently across scenarios (see Supplementary Table 1).

### Prediction of response rationale

#### **Fabry’s scenario**

In each model, the reference category (default response choice represented by the intercept) was self-involving, as this was the most frequently chosen GC response for the scenario. The final logistic regression models for predicting the five rationales are shown in Table 4. Participants who chose the influencing instead of self-involving GC response (*p*=.003, *OR* = 3.29) or were single (*p*=.03, *OR* = 2.05) were more likely to provide *Understanding* as their rationale. Choosing the content (*p*<.001, *OR* = 0.10), affect (*p*=.001, *OR* = 0.09), or question GC response (*p*<.001, *OR* = 0.02) instead of self-involving all significantly predicted lower likelihood of a *Problem Solving* rationale. Selecting the content (*p*=.02, *OR* = 15.39) or question GC response (*p*=.03, *OR* = 18.02) instead of self-involving increased the likelihood of a *Focusing on the Patient* rationale, while having children decreased the likelihood of *Focusing on the Patient* (*p*=.008, *OR* = 0.11). Having children (*p*=.04, *OR* = 2.58) increased the likelihood of using a *Validating* rationale.

**Table 4:** Final logistic regression models predicting rationales for each scenario

Variable	AIC	*R* ^2^	b	SE	Wald’s z	*p*	OR [95% CI]
Fabry Disease							
*Understanding*	262.73	0.15					
Intercept			-3.26	1.27	-2.58	0.01*	
Content Response			0.66	0.40	1.67	0.10	1.93 [0.89, 4.20]
Question Response			1.20	0.62	1.94	0.05	3.31 [0.99, 11.07]
Influencing Response			1.19	0.40	3.01	0.003**	3.29 [1.52, 7.15]
White			0.64	0.34	1.87	0.06	1.89 [0.97, 3.68]
Single			0.72	0.33	2.19	0.03*	2.05 [1.08, 3.89]
Family History of Fabry			1.07	0.62	1.75	0.08	2.93 [0.88, 9.78]
*Problem Solving*	155.49	0.58					
Intercept			-0.88	0.72	-1.21	0.22	
Content Response			-4.66	1.07	-4.36	< 0.001***	0.01 [0.00, 0.08]
Affect Response			-2.36	0.74	-3.20	0.001**	0.09 [0.02, 0.40]
Question Response			-18.17	970.67	-0.02	0.99	0 [0, 0]
Influencing Response			-4.14	0.80	-5.18	< 0.001***	0.02 [0.00, 0.08]
Age			0.03	0.02	1.78	0.08	1.03 [1.00, 1.07]
Have Heard of GC			0.68	0.45	1.52	0.13	1.97 [0.82, 4.72]
*Sympathizing*	101.61	0.22					
Intercept			-2.79	0.73	-3.81	< 0.001***	
Content Response			-1.27	0.78	-1.63	0.10	0.28 [0.06, 1.29]
Affect Response			-17.75	2793.03	-0.01	0.99	0 [0, 0]
Influencing Response			-17.97	1593.42	-0.01	0.99	0 [0, 0]
White			1.42	0.78	1.83	0.07	4.14 [0.90, 19.02]
*Focusing on the Patient*	80.39	0.31					
Intercept			-2.34	1.23	-1.90	0.06	
Content Response			2.73	1.15	2.38	0.02*	15.39 [1.63, 145.73]
Affect Response			2.65	1.54	1.72	0.08	14.21 [0.69, 290.53]
Question Response			2.89	1.34	2.15	0.03*	18.02 [1.29, 251.52]
Influencing Response			1.66	1.19	1.40	0.16	5.28 [0.51, 54.61]
Have Children			-2.25	0.85	-2.64	0.008**	0.11 [0.02, 0.56]
Familiarity with GC			-0.87	0.53	-1.65	0.10	0.42 [0.15, 1.18]
*Validating*	167.08	0.07					
Intercept			-2.14	0.41	-5.27	< 0.001***	
Content Response			-0.88	0.65	-1.36	0.17	0.42 [0.12, 1.47]
Question Response			-1.35	1.06	-1.27	0.20	0.26 [0.03, 2.08]
Have Children			0.95	0.46	2.05	0.04*	2.58 [1.04, 6.40]
Cleft Scenario							
*Understanding*	240.63	0.29					
Intercept			-0.57	0.40	-1.40	0.16	
Affect Response			-2.29	1.10	-2.08	0.04*	0.10 [0.01, 0.88]
Question Response			-1.62	0.49	-3.34	< 0.001***	0.20 [0.08, 0.51]
Influencing Response			-2.29	0.66	-3.47	< 0.001***	0.10 [0.03, 0.37]
White			0.86	0.37	2.34	0.02*	2.37 [1.15, 4.87]
Income between $50–100 K			0.88	0.41	2.17	0.03*	2.41 [1.09, 5.33]
Income > $100K			0.34	0.42	0.83	0.41	1.41 [0.62, 3.19]
*Sympathizing*	136.29	0.22					
Intercept			-1.09	0.41	-2.67	0.01*	
Content Response			-1.84	0.69	-2.68	0.007**	0.16 [0.04, 0.61]
Affect Response			-17.77	2118.56	-0.01	0.99	0 [0, 0]
Question Response			-1.29	0.79	-1.64	0.10	0.27 [0.06, 1.29]
Influencing Response			-17.83	1377.83	-0.01	0.99	0 [0, 0]
Single			1.01	0.50	2.03	0.04*	2.75 [1.03, 7.31]
Have Heard of GC			-0.73	0.47	-1.54	0.12	0.48 [0.19, 1.22]
*Focusing on the Patient*	92.96	0.44					
Intercept			-3.50	0.74	-4.71	<0.001***	
Content Response			-16.90	1491.08	-0.01	0.99	0 [0, 0]
Question Response			2.65	0.57	4.64	< 0.001***	14.12 [4.62, 43.15]
Influencing Response			-16.73	2310.98	-0.01	0.99	0 [0, 0]
White			1.02	0.73	1.40	0.16	2.77 [0.67, 11.48]
*Validating*	204.47	0.18					
Intercept			-0.90	0.54	-1.66	0.10	
Question Response			-1.01	0.60	-1.68	0.09	0.37 [0.11, 1.18]
White			-0.99	0.42	-2.33	0.02*	0.37 [0.16, 0.86]
Associate’s Degree			-0.19	0.57	-0.33	0.74	0.83 [0.27, 2.54]
Bachelor’s Degree			-1.21	0.48	-2.50	0.01*	0.30 [0.12, 0.77]
Graduate Degree			-2.18	0.71	-3.09	0.002**	0.11 [0.03, 0.45]
Income between $50–100 K			0.14	0.49	0.29	0.77	1.16 [0.44, 3.02]
Income > $100K			1.36	0.53	2.58	0.009**	3.88 [1.38, 10.86]
Have Heard of GC			1.34	0.46	2.88	0.004**	3.81 [1.53, 9.45]
Offered Genetic Testing			-0.76	0.45	-1.71	0.09	0.47 [0.19, 1.12]

*Note. R*^2^ reported is Nagelkerke’s *R*^2^; reference GC response is the self-involving response; *p < .05; **p < .01; ***p < .001

#### **Cleft scenario**

The final logistic regression model for predicting the four rationales in the Cleft scenario are presented in Table 4. White participants (*p*=.02, *OR* = 2.37) and those with an income between $50–100 K a year (*p*=.03, *OR* = 2.41) were more likely to provide an *Understanding* rationale, while those selecting the affect (*p*=.04, *OR* = 0.10), question (*p*<.001, *OR* = 0.20), or influencing GC response (*p*<.001, *OR* = 0.10) were less likely. White participants were more likely to provide a *Sympathizing* rationale (*p*=.04, *OR* = 2.75), while those who selected the content GC response were less likely to do so (*p*=.007, *OR* = 0.16). Those who chose the question GC response were more likely to provide a *Focusing on the Patient* rationale (*p*<.001, *OR* = 14.12). Participants with an income >$100K (*p*=.009, *OR* = 3.88), and those who had previously heard of genetic counseling (*p*=.004, *OR* = 3.81) were more likely to provide a *Validating* rationale, while those who identified as white (*p*=.02, *OR* = 0.37), had a Bachelor’s degree (*p*=.01, *OR* = 0.30), or had a Graduate degree (*p*=.002, *OR* = 0.11) were less likely to do so.

## Discussion

This study aimed to examine what types of genetic counselor responses proxy patients perceive as most empathic and why. Overall, results indicate variation in preferences of GC empathy statements, as well as rationale for those preferences. Kao (2010) found genetic counselors’ empathy responses varied widely in their content, length, and skill, suggesting there is not a prescribed way of demonstrating empathy. Our findings suggest patients may differ in what they want in an empathy response (received empathy) and there is not a one size fits all approach to communication of empathy (expressed empathy). Previous researchers found proxy patients’ perceived skillfulness of genetic counselors’ self-disclosure responses varied, and they recommended genetic counselors tailor their responses to the specific patient and context (Greve et al. 2019).

While every GC response was endorsed by one or more proxy patients as empathic, those that included a self-involving statement of “I’m sorry” were chosen most often in both scenarios. These statements initiate what some theorists and researchers consider to be a statement of sympathy (e.g., Clark 2010; McCarthy Veach et al. 2019). A sympathetic statement has a different intent (expressing compassion for the client’s difficult circumstances) than an empathy statement (expressing an understanding of the client)(Clark 2010; McCarthy Veach et al. 2019). Clark (1987) found sympathy is involved in social etiquette; therefore, participants’ preference may be due to social expectations. Other authors (e.g., Bachelor & Freedheim, 1988) regard “I’m sorry” as a form of shared empathy in which the counselor expresses their personal reaction. Shared empathy may resonate with participants due to a desire to know what the counselor thinks, and it therefore feels most empathic. Additionally, Kashmola-Perez et al. (2021) in a study of self-involving statements in genetic counseling, found that the genuineness conveyed through statements like “I’m sorry” can communicate empathy to the patient. Further studies are needed to assess whether patients receive it as such or view it as social etiquette/politeness.

Both GC responses used in the present study, however, are more complex than a simple sympathy statement. The genetic counselor in the Fabry disease scenario followed “I’m sorry,” with a self-disclosure statement expressing a desire to help the patient find a solution. In the Cleft scenario, the counselor followed “I’m sorry,” with a content statement reflecting the patient’s difficult situation. It is unknown what aspects of the genetic counselor responses specifically influenced participants’ preferences, though we unpack this further in the next section on participants’ rationales for their choices.

### Respondents’ rationales for their preferred GC responses

Inductive content analysis of participants’ rationale for choosing their selected GC response generated six categories. Overall, *Understanding* was the most prevalent rationale for both the Fabry and Cleft scenarios and was provided at least once for each scenario. Empathy at its core involves sensing and understanding a person’s experience and communicating that understanding to them (McCarthy Veach et al. 2019). As understanding is a part of empathy, the term “understanding” was used as in the description of empathy in this study which may have influenced participants’ use of this term in their rationales.

Returning to the issue of sympathy vs. empathy discussed above, we can start to unpack what participants focused on when they made their selection. The proportion of participants classified as using a *Sympathizing* rationale was relatively low (9.1% for Fabry, 12.1% for Cleft), and these individuals overwhelmingly selected the GC response containing “I’m sorry” (83.3% for Fabry, 79.2% for Cleft). Among those who selected the “I’m sorry” GC response, however, there was a much smaller percentage (17.4% for Fabry, 21.4% for Cleft) who had a *Sympathizing* rationale. This suggests participants were largely drawn to these GC responses, not for the sympathy aspect, but for the other portion (self-disclosure for Fabry and content statement for cleft). Indeed, the proportion of people who selected the “I’m sorry” GC response for Fabry disease with a *Problem Solving* rationale was much higher (66.3%) which likely indicates the preference for the component of the GC response “I’d like to help you find a…” rather than the component of “I’m sorry.” We cannot, however, determine the degree to which the written rationale provided by participants accurately reflects wholly the influences of the counselor statements, or the degree to which the components of these statements interact. Further research should continue to explore the impact of components of empathy statements, both singly and in combination.

The overall pattern of rationales indicates participants had a variety of reasons for selecting their GC response, which further suggests individual variation in patient perceptions of and reactions to genetic counselor expressions of empathy (expressed and received empathy, respectively). This is consistent with findings from psychotherapy and nursing where clients/patients assess empathy in a multifaceted manner in regard to how client’s perception of empathy (e.g., is it accurate? does it emotionally resonate with me? ) or what component of empathy is interpreted (e.g., empathy as a trait or a communication process) (e.g., Bachelor & Freedheim, 1988; Kunyk and Olson 2001).

While this study only included two genetic scenarios, findings in proxy patients’ rationales may suggest that what patients want in an empathy GC response differs depending on several contextual factors. For example, there may be differences based on their genetic situation (in this study Fabry vs. Cleft scenario). Fabry disease is an invisible illness, as one cannot determine if a person is affected merely by looking at them. In contrast, Cleft lip and palate is a more well-known and highly visible condition; and perhaps that is why proxy patients were more likely to want validation. As noted by Tomozawa et al. (2025), patient’s perception of empathy may be influenced by a patient’s context prior to empathic experiences, often related to their expectations, prior experiences, or needs from genetic counseling. Additionally, there are differences in the scenarios in the temporal and emotional context that may have influenced proxy patients’ rationales. The Fabry disease scenario described a more chronic, ongoing experience of physical pain, whereas the cleft scenario involved a past memory or experience that involved another’s opinion and reaction that caused psychological pain or harm. Thus, the desired empathy response from the GC may differ based on the desired response to these contextual differences. Again, assessment of the patient’s context and subsequent tailoring of empathy GC responses remain important.

### Prediction of response rationale

Predictors of response rationale should be used cautiously to provide an idea of what patients think best fits the provided definition of empathy. While these predictors may be helpful in gaining a deeper understanding of the patient for genetic counselors to tailor an empathy response, which predictors were significant varied across scenarios and between rationales, again signaling context is important when considering empathy. This is illustrated by the differences noted in how rationales predicted GC responses between the Fabry disease and Cleft scenarios with considerable variation in the Fabry disease scenario (see Table 2) and more balance between GC responses and demographic variables in the Cleft scenario, though there was again little consistency in predictors across rationales.

Overall, while there was at least one GC response that significantly predicted each rationale (with the exception of *Sympathizing* in the Fabry disease scenario), there was little consistency across rationales and several GC responses predicted multiple rationales. At this time, the data suggest relatively low consensus in terms of how to approach selecting the most empathic response. Similarly, few demographic variables were significant predictors of rationales, again suggesting demographic variables cannot be used to make assumptions of the patient’s deeper needs. It is critical that the genetic counselors assess the patient’s underlying feelings and needs to provide an effective empathy response. This underscores the importance of the relationship between the patient and counselor, which is consistent with the REM, where the relationship between counselor and patient is the critical component (McCarthy Veach et al. 2007). This relationship, based on good communication and established bond, transcends demographic categories.

### Study limitations

This study used hypothetical genetic counseling scenarios and proxy patients which may limit the extent to which these findings are generalizable to actual genetic counseling sessions. Survey respondents were all female-identifying (which was intentional given the prenatal scenarios used in this study) but admittedly, this limits the generalizability of results to a specific population. Participants were instructed to choose the most empathic response without additional instructions, so social desirability or personal preference may have influenced chosen response rather than sole characteristics of empathy. Additionally, a brief scenario does not capture the entirety of genetic counseling goals and counselor-patient interactions during a session. As a majority of the present sample reported little familiarity with genetic counseling, they likely lacked an understanding of what would “come before and after” the types of genetic counselor responses in the two scenarios, though degree of familiarity did not significantly predict the use of any rationale. Of note, it is not uncommon for patients to be unfamiliar with genetic counseling and thus, this unfamiliarity could also bolster ecological validity of the study findings.

The decision to model the data to predict participants’ rationales certainly led to different conclusions than if we predicted these choices of counselor responses. We made this decision because we judged the rationales to be more consistent across samples and across the infinite possible empathy statements that could come from the counselor. Predicting GC responses based on rationales and demographic variables may have uncovered a different set of relationships. We decided to evaluate all models with an alpha level of 0.05 despite conducting multiple tests due to the exploratory nature of our study. As replication is needed to confirm the results of this study and extend them to other statements and contexts, we judged a Type II error to be more problematic in this case because potential variables of interest could be discarded prematurely. This does, however, make the likelihood of a Type I error more likely than if familywise error corrections had been applied at the per-comparison level.

The use of written genetic counselor empathy responses as opposed to in person responses may have further limitations. The statements lack verbal (e.g., tone) and non-verbal (e.g., body language) cues. As noted recently by Meredith et al. (2025) there may be an interplay of non-verbal cues and empathy responses that impact the effectiveness of how empathy is perceived. Another limitation is that the genetic counselor empathy responses derived from Kao (2010) were exemplars of specific categories, and some were more complex than others (i.e., involving different types of statements). They were chosen as the most prevalent response domains in Kao’s study and admittedly do not represent all possible variations of genetic counselor-defined empathy statements. We chose to prioritize external validity in this study in that we wanted real statements submitted by genetic counselors as the stimulus rather than strictly controlling length and type of the statement. We recognize this makes interpretation more challenging and emphasize the need for additional research that explores the trade-offs between internal and external validity. Lastly, the definition of empathy used in this study contained the word understanding, which may have steered some participants toward an *Understanding* rationale for their response preferences.

### Practice implications

The results indicate there is not a single preferred empathy response for all, or even the majority of, proxy patients. The findings further suggest an ongoing assessment of the patient’s feelings and needs facilitates an effective empathic response. This fits with Barrett-Lennard’s (1981) cyclical model of empathy which entails empathic resonance (assessment of the patient), expressed empathy (communication of empathy), and received empathy (patient’s perception of empathy). Continual check-ins with patients throughout the GC session support this ongoing assessment from start to finish of the patient’s GC experience.

The REM asserts the genetic counselor-patient relationship is central to the genetic counseling process and that empathy is integral to this relationship (McCarthy Veach et al. 2007). Differences in proxy patients’ perceptions of empathic statements in the present study suggest that in order to build this relationship, counselors must recognize individual patient needs and tailor their empathy GC responses for each patient and not base their assessment on demographic variables. Building rapport through contract and mutual agenda setting will contribute to the GC-patient relationship that can then foster such tailoring.

Every GC response was preferred by at least one proxy patient in the present study. Thus, these responses may be appropriate for certain patients in certain contexts. For some patients, validation may be fitting; for others, reflections that exhibit active listening of content or affect may be warranted. Some authors (e.g., McCarthy Veach et al. 2019) assert that when a response conveys some evidence of the counselor’s understanding of patient feelings and experience, “exact wording” may be less critical; rather, *how* the genetic counselor expresses empathy may be more impactful than *what* the counselor actually says. While this study did not focus on non-verbal behaviors, a combination of verbal and non-verbal behaviors conveying an attitude of respect and non-judgment may be optimal (e.g., eye contact, leaning in, active listening).

### Research recommendations

Further research involving proxy patients’ perceptions of genetic counselor empathy responses could include measures of the participants’ ability to empathize with the hypothetical patient. Further studies could more accurately simulate real sessions such as video recorded or hypothetical in-person sessions or ideally actual patients. Studies of that type would allow assessment of both verbal and nonverbal cues and allow assessment of the impact of multiple empathy responses within a session. A promising method for studying the interplay of these components of empathy n genetic counseling is interpersonal process recall (IPR; Tomozawa et al. 2023). Future IPR studies could involve proxy or actual patients reviewing segments of videotaped sessions they participated in to discuss their perceptions of helpful and hindering genetic counselor empathy behaviors (verbal and non-verbal) in a full genetic counseling session (Gale et al. 2010). This would allow for a more systematic examination of the overall genetic counseling process and components that influence genetic counselors’ assessment and approach to empathy and patients’ interpretation of GC empathy. Studies involving other hypothetical scenarios representing varied genetic counseling specialties and additional genetic counselor empathy responses would help to replicate and extend the present results.

## Conclusion

This study provides initial evidence regarding proxy patients’ perceptions of genetic counselor-defined empathy responses. Patients differed in their choice of the most empathic genetic counselor responses in each of two scenarios, as well as in their rationales for their preferences. The major categories found reflected in their rationales were *Understanding*,* Sympathizing*,* Problem Solving*,* Focusing on the Patient* and *Validating.* Few demographic variables were significant predictors of proxy patients’ rationales and no demographic variables were consistent predictors across rationales. These findings highlight that empathy is an ongoing process of discernment and communication and suggest the importance of genetic counselors responding to the patient in the moment and not relying on phrases or scripts, as patients will vary in their preferences regarding expressed empathy.

## Data Availability

Data for this study are available upon request from the corresponding author. All data obtained during this study that are relevant to the major research questions are reported in the published article, tables, and supplemental material.
